# Supplementary material for: Isolation and Characterization of the Genes Involved in the Berberine Synthesis Pathway in Asian Blue Cohosh, Caulophyllum robustum
Source: Plants (Basel). 2023 Mar 28;12(7):1483. doi: 10.3390/plants12071483 (PMC10096549; doi:10.3390/plants12071483)
Supplement: Supplementary file 1 [file plants-12-01483-s001.zip › Supplement table S1, S2, S3, S4.Berberine synthesis genes.pdf]

Supplementary Table S1. Number of reads and read length before and after processed

	Raw data		Cleaned data	
Sample	Number of reads	Length (bp)	Number of reads	Length (bp)
Caulophyllum1 (fruit)	58,937,132	8,899,506,932	53,745,338 (91.19)*	7,408,922,298 (83.25)
Caulophyllum2 (leaf)	70,856,856	10,699,385,256	64,276,390 (90.97)	8,866,906,225 (82.87)
Caulophyllum3 (stem)	67,616,396	10,210,075,796	61,966,482 (91.64)	8,460,162,998 (82.86)
Total	197,410,384	29,808,967,984	179,988,210 (91.17)	24,735,991,521 (82.98)

Note: The numbers in brackets are percent after cleaned from the raw data.

Supplementary Table S2. Mapping Information of the clean reads.

	Fruit	Leaf	Stem
Number of total reads	53,745,338	64,276,390	61,966,482
Number of Mapped PE Reads	37,095,516	46,280,136	43,994,548
% of mapped PE Reads number	72	69	71

Supplementary Table S3. De novo assembly results.

	No. of contigs	Length of contigs	Minimum length	Maximum length	Average length
Trinity	252,149	197,484,773	180	16,539	783
CD-HIT-EST	225,623	167,006,458	189	16,539	740
Mapping Filter (10> reads)	175,172	153,608,750	189	16,539	877
TransDecoder.LongOrfs	96,030	67,303,215	297	14,427	701
TransDecoder.Predict	58,659	91,486,519	297	16,539	1,338
Redundance remove	41,094	54,964,984	297	16,539	1,338
Final	41,094	54,964,984	297	16,539	1,338

Supplementary Table S4. Number of expressed genes in different tissues.

	Leaf	Fruit	Stem
0	3,986	5,497	4,486
>0	37,108	35,597	36,608
