# Supplementary material for: Isolation and Characterization of the Genes Involved in the Berberine Synthesis Pathway in Asian Blue Cohosh, Caulophyllum robustum
Source: Plants (Basel). 2023 Mar 28;12(7):1483. doi: 10.3390/plants12071483 (PMC10096549; doi:10.3390/plants12071483)
Supplement: Supplementary file 1 [file plants-12-01483-s001.zip › Supplementary Figure S1.pdf]

6OMT GroupI

```
                   I                      II
Bkor6OMT3       SALINECKDKFQGINSLVDVGGGTGTAARNLVKAFPHLKCTVYDLPHVIADSPVYPEITQ      232
Cro6OMT4        SALVNECKGVFKGVKTLVDVGGGTGTAVMNIAKAFPSIQCSVYDLAHVIADSNAFPEINR      233
Cro6OMT3        SAFLNKCKDKFIGITSLVDVAGGTGTAVWNIAKAFPHIKCTLYDLPHVIADSPAYPEIDR      229
Cro6OMT5        SALVNEGKDKFIGITSLVDVGGGTGTAVWNIAKAFPHIKCTVFDLPHVIADSVAYPEINR      230
Cro6OMT6        SALVNEGKDKFIGITSLVDVGGGTGTAVWNIAKAFPHIKCTVFDLPHVIADSVAYPEINR      89
                **::*: *. * *:.:****.******. *:.**** ::*:::** ****** .:*** :

                 III                                         IV
Bkor6OMT3       VPGDMFKVIPYADAILMKCILHDWGDKECIEILKKCKEALPKEGGKVIIVDIVLDVKSEH      292
Cro6OMT4        VAGDMFEHIPNADAILMKCILHDWSDEECIQILKKCKKAVPKNGGKVIIVDIVLDVESQH      293
Cro6OMT3        VAGDMFKYIPSADAILMKYILHDYDDEKCIQILKKCKKAVSEDGGKVIIIDIVLNVDSKH      289
Cro6OMT5        VAGDMFNYIPSADAILMKSILHGFDDAECTQILKKCKKAVPKDGGKVIVIDIVLNGESEH      290
Cro6OMT6        VAGDMFNYIPSADAILLKYILHDFDDEECTQILKKCKKAVPKDGGKIIIIDIVLNVESEH      149
                * ****: ** *****:* ***.:.* :* :******:*: ::***:*::****: .*:*

                    V
Bkor6OMT3       PYTKLRLTLDVDMMLNTGGGKERTEEEWKKLIQDAGFKSYNITQISALQSVIEAFPY         349
Cro6OMT4        PYTKMRLTLDLDMMLNTGGGKERTEEEWKKLIYAAGFTSYNITQISAAQSVIEAFPY         350
Cro6OMT3        PLTKFRVSSDLDMMLTTGGGKERTKEEWKKLFKAAGYKGYNITEISAIQSVIEAFPY         346
Cro6OMT5        PYTKMRVTMDMDMMIATEGGKERTEEEWKKLFDAVGFRSYKISQISALQSVIEAFPY         347
Cro6OMT6        PYTNMRVTMDIDMMIGTGGGKERTEEEWKKLFGAAGFRSYNISQISALQSVIEAFPY         206
                * *::*:: *:***: * ******:******:  .*: .*:*::*** *********
```

6OMT Group II

```
                            I                          II
Bkor6OMT1       SHTSMVTDAVVKGFEKDHVLDGMTSLIDVGGSTGVAARAIAKAFPE-VKCAVFDLPHVIA      218
Bkor6OMT6       SHTSMVTDAVVKGFEKDHVLDGMTSLIDVGGSTGVAARAIAKAFPE-VKCAVFDLPHVIA      218
Bkor6OMT2       SHTMFVIPAVVGGLMKSKILDDVKSLVDVGGSTGAAAKGIADGFPH-VKCFVMDLAHVIE      224
Bkor6OMT7       SHTMFVIPAVVGGLMKSKILDDVKSLVDVGGSTGAAAKGIADGFPH-VKCFVMDLAHVIE      224
Cro6OMT2        SHTKCMVPALVTGLVKEKVLDDVTSLVDVGGSEGIAAKAIKEIASH-VKCVVMDLAHVIE      230
Cro6OMT1        SHTKCMVSALVVGLTKERVLDDVTILVDVGGSEGVASKAIVDAASH-VKCIVMDLSHVIE      229
Bkor6OMT4       SHTMCMVPALVSGLTKENVLADVASLMDVGGSEGIASKAVIQVVSHIVKCIIMDLAHVID      146
Bkor6OMT5       SHTMCMVPALVSGLTKENVLADVASLMDVGGSEDWISWL---------------------      125
                ***  :  *:* *: *..:* .:  *:***** .  :

                           III                                 IV
Bkor6OMT1       TAPECPEVTRIEGDVFVSLPKTDLVFMKSVLHDWGDEDCVKILNKCKEAIADSKQGKIVL      278
Bkor6OMT6       TAPECPEVTRIEGDVFVSLPKTDLVFMKSVLHDWGDEDCVKILNKCKEAIADSKQGKIVL      278
Bkor6OMT2       SVPKDGGLNFVAGDMFSFIPKADAIFLKSVLHNYDDDVCLKILAKCKEAIPL-TKGKVIL      283
Bkor6OMT7       SVPKDGGLNFVAGDMFSFIPKADAIFLKSVLHNYDDDVCLKILAKCKEAIPL-TKGKVIL      283
Cro6OMT2        TVPKDARLEYLAGDMFASIPQTDAVLLKSVLHNYGDDRGLEILGKCKVAIPA-TKGKVIL      289
Cro6OMT1        SVPKDPRLDFLAGDMFAFVPQTDAVLLKSILHNYDDDHCVKILTMCKEAIHS-TRGKVII      288
Bkor6OMT4       TVPNDPRLDFLAGDMFDFVPKADAVLLKSILHNYDDENCLKILTKCKEAIPT-KGGKVVI      205
Bkor6OMT5       -------------VTFDFVPKADAVLLKSILHNYDDENCLKILTKCKEAIPT-KGGKVVI      171
                               *  :*::* :::**:**::.*:  ::**  ** **   . **:::

                               V
Bkor6OMT1       VEIVMDAVSTDDEFKSAKLGMEMDMLVTVAGKERSEQEWQSLFKAAGYSRYHITPIVAIE      338
Bkor6OMT6       VEIVMDAVSTDDEFKSAKLGMEMDMLVTVAGKERSEQEWQSLFKAAGYSRYHITPIVAIE      338
Bkor6OMT2       VEIVVDVE-TLPEFSSARCCMDMEMML-MGGKERTKQEWVDLLDKAGFSHHEIIPVMA--      339
Bkor6OMT7       VEIVVDVE-TLPEFSSARCCMDMEMML-MGGKERTKQEWVDLLDKAGFSHHEIIPVMA--      339
Cro6OMT2        VEIVLETE-NLAEFAPGRLGVDMQMIC-MGGKERTKQEWECLLHKAGFTLHNIIPIMANE      347
Cro6OMT1        VEIIIDTE-NLQEFSSARLGLDMEMLC-MGGKERTKQEWKCLLDKVGFSHHDTIPIRAIE      346
Bkor6OMT4       VEIVIDTQ-NLPEFSSARLGLDMEMIC-MGGQERTKQEWATLLHKAGFDHHTIIPIRAIE      263
Bkor6OMT5       VEIVIDTQ-NLPEFSSARLGLDMEMIC-MGGQERTKQEWATLLHKAGFDHHTIIPIRAIE      229
                ***:::.  .  **  .:  ::*:*:  :.*:**::***  *:. .*:  :   *: *
```

Supplementary Figure S1. Sequence comparison of the group 1 and group II of 6OMTs of *C. robustum* and *B. koreana.* The conserved motif sequences are highlighted with red. The motifs I and IV are S-adenosyl-L-methionine binding (conserved region I) and metal binding (conserved region IV) sites, respectively.
